# *Scheffersomyces spartinae* Fungemia among Pediatric Patients, Pakistan, 2020–2024

**DOI:** 10.3201/eid3108.241604

**Published:** 2025-08

**Authors:** Kauser Jabeen, Joveria Farooqi, Lacy M. Simons, Judd F. Hultquist, Ramon Lorenzo-Redondo, Charlesnika T. Evans, Erica M. Hartmann, Mohammad Hanif, Zahra Hasan, Syed Faisal Mahmood, Javaria Ashraf, Hassan Ghayas, Sadaf Zaka, Noureen Saeed, Sayed Ali Raza Shah Bukhari, Mehreen Arshad, Larry K. Kociolek, Sameer J. Patel, Rumina Hasan, Egon A. Ozer

**Affiliations:** Aga Khan University, Karachi, Pakistan (K. Jabeen, J. Farooqui, Z. Hasan, S.F. Mahmood, J. Ashraf, H. Ghayas, S. Zaka, N. Saeed, S.A.R. Shah Buhari, M. Arshad, R. Hasan); Northwestern University Feinberg School of Medicine, Chicago, Illinois, USA (L.M. Simons, J.F. Hultquist, R. Lorenzo-Redondo, C.T. Evans, E.M. Hartmann, E.A. Ozer); Northwestern University Havey Institute for Global Health, Chicago (L.M. Simons, J.F. Hultquist, R. Lorenzo-Redondo, E.A. Ozer); Edward Hines Jr. VA Hospital, Hines, Illinois, USA (C.T. Evans); Northwestern University McCormick School of Engineering, Evanston, Illinois, USA (E.M. Hartmann); National Institute of Child Health, Karachi (M. Hanif); Ann & Robert H. Lurie Children’s Hospital of Chicago, Chicago (M. Arshad, L.K. Kociolek, S.J. Patel); London School of Hygiene and Tropical Medicine, London, UK (R. Hasan)

**Keywords:** Scheffersomyces spartinae, fungi, antimicrobial resistance, emerging pathogen, yeast, fungemia, Clavispora lusitaniae, neonatal infection, pediatric infection, United States, Pakistan

## Abstract

Prevalence of emerging fungal infections is increasing, particularly among immunocompromised persons, children, and older persons. We report 108 cases of *Scheffersomyces spartinae* infection in pediatric patients from Karachi and other cities in Pakistan, of which 107 were identified from blood cultures. Cultures were initially misidentified as *Clavispora lusitaniae* by a biochemical assay before speciation as *S. spartinae* by whole-genome sequencing. All isolates were from children <12 years of age, and >69% were from children <1 month of age. Isolates were genetically distinct across regions of Pakistan; however, genetic diversity was low in isolates from patients in Karachi and nearby Nawabshah and had median differences of just 9 pairwise nucleotide variants. This study demonstrates *S. spartinae* is a potentially emerging pathogen in neonates and young infants in Pakistan. The findings highlight the limitations of phenotypic identification for detecting emerging fungal infections and underscore the value of molecular identification approaches.

Globally, fungi continue to emerge as serious threats to human health, marked by an increase in incidence of invasive fungal infections and deaths, the growing burden of cutaneous and allergic fungal diseases, and the rapid emergence of antifungal resistance in fungi pathogenic to humans (*1*). The number of fungi associated with invasive disease is rising as new pathogenic fungi emerge and modern sequencing and analysis approaches drive insights into fungal taxonomy (*2*,*3*). Emergence of new pathogenic species and increased infections caused by previously rare or region-specific pathogens might be attributed in various cases to climate change (*4*–*7*), more frequent or severe natural disasters (*8*,*9*), expanding host populations (*10*–*12*), increased use of antifungal agents in clinical or industrial practice, or other unknown epidemiologic drivers.

Considering the immense diversity of fungi and the emergence of new pathogens, ongoing epidemiologic and molecular surveillance of fungal infections is essential. Current phenotypic and biochemical methods do not reliably identify rare or emerging fungal pathogens, and misclassification can occur (*13*–*15*). Molecular approaches, such as internal transcribed spacer (ITS) sequencing, and genomic approaches, such as whole-genome sequencing (WGS), can provide greater specificity for distinguishing new or uncommon fungal infections; however, those methods might not be available to clinical microbiology laboratories in some resource-limited settings (*16*).

In late 2022, the Aga Khan University Hospital (AKUH) clinical mycology laboratory in Karachi, Pakistan, noticed increased cases of yeast, biochemically identified as *Clavispora lusitaniae*, cultured from the blood of infants admitted to neonatal intensive care units. Most isolates were subsequently identified by WGS as *Scheffersomyces spartinae*, an environmental yeast with high tolerance to temperature, pH, and salinity changes (*17*,*18*). *S. spartinae* has been isolated from brackish waters, marshes, and oceans and can survive temperatures of 5°C–35°C, pH of 3–9, and salinity of 0–60 g/L NaCl (*19*,*20*). We conducted genomic analyses of available isolates to identify epidemiologic characteristics of *S. spartinae* infections in Pakistan.

## Methods

### Study Setting and Duration

The study was performed at AKUH during 2020–2024. The AKUH clinical microbiology laboratory received specimens from 315 satellite collection units in 99 cities and towns across all 4 provinces of Pakistan. Specimens were sent at the primary physician’s request; the laboratory had no control over specimen ordering. 

### Isolate Collection

The AKUH clinical microbiology laboratory followed a standardized approach for processing fungal cultures and selecting specimens for archiving. The laboratory received and cultured blood samples in a BacT/ALERT continuous monitoring system (bioMérieux) for 5 days. If samples were flagged positive, results of Gram stain from the broth determined the choice of solid media for subculture. Specimens with yeast on Gram stain were inoculated on BD BBL sheep blood agar, BD BBL Saboraud’s dextrose agar, and BD BBL CHROMagar chromogenic agar plates (all from Becton, Dickinson and Company) and incubated at 35°C–37°C. In addition, the laboratory staff struck yeast on BiGGY agar and Corn Meal Agar with Tween 80 (all Oxoid) for morphological identification, then performed biochemical identification by using the VITEK 2 YST ID card system (bioMérieux). 

Antifungal susceptibility was determined by using the Sensititre YeastONE system (Thermo Fisher Scientific). Laboratory staff regularly reviewed sample processing records and selected isolates resistant to fluconazole, echinocandins, or amphotericin B and uncommon or rare yeast isolates for banking. Selected isolates were recultured on potato dextrose agar and incubated at 37°C for 48 hours. Staff transferred colonies from pure cultures to vials of 50% glycerol phosphate broth and banked isolates at −80°C. 

### Retrospective Data Review

We reviewed the laboratory database to identify all cases of *C. lusitaniae* in blood cultures from 2015–2024. We retrieved patient age, sex, location of residence, and contact details from the clinical laboratory’s integrated laboratory information system. For most cases, the contact details were for patients or their guardians and not for the treating physicians. We obtained informed consent from the patients or their guardians by telephone in the presence of a witness before obtaining medical histories. We recorded information in a database on REDCap. The AKUH ethical review committee provided ethical review and approval (study nos. 2022-6798-20372 and 2019-0438-2659).

### Whole-Genome Sequencing

We inoculated banked isolates in sterile brain heart infusion broth for overnight incubation at 37°C and subcultured on Sabouraud’s dextrose agar for DNA extraction. We performed cell lysis from colonies by using glass beads in lysis buffer and proteinase K and 2 cycles of vortexing at 3,000 rpm for 1 minute each. We then incubated cells at 56°C for 15 minutes. We used the QIAquick DNA Minikit (QIAGEN) to extract genomic DNA from the lysate using the default protocol, eluted in AE buffer, and either stored at −20°C for <2 weeks before sequencing or stored at −80°C if sequencing was not performed within 2 weeks. Sequencing was performed at Aga Khan University (AKU) or Northwestern University (NU; Chicago, Illinois, USA). We used the Speed Vac vacuum concentrator (Thermo Fisher Scientific) to dry extracted DNA specimens before shipping to NU at ambient temperature. NU performed WGS by using the NovaSeq X or MiSeq platforms (Illumina) after library preparation with the plexWell Library Prep Kit (SeqWell). AKU sequenced a subset of the specimens on the Illumina MiniSeq platform after library preparation using the Nextera XT Kit (Illumina). 

We trimmed Illumina read sequences by using fastp version 0.23.2 and performed de novo assembly by using SPAdes version 3.9.1. We removed contigs <200 bp or that had <5× mean read coverage. We used BUSCO and the saccharomyces_odb10 database (*21*) to assess completeness of genome assembly. We performed species identification by extracting ITS sequences from draft assemblies by using in silico PCR and previously described primer sequences ITS5 and ITS4 (*22*). We compared resulting sequences against the National Center for Biotechnology Information (NCBI) Fungal ITS RNA sequence database (BioProject accession no. PRJNA177353) using BLAST (*23*). We generated ITS sequence alignments by using MAFFT (*24*), and generated neighbor-joining trees by using RapidNJ (*25*).

### Long-Read Sequencing

We randomly selected 1 isolate, 128-CS, for long-read sequencing on the MinION sequencer at AKU by using a Flongle Flow Cell R10.4.1 after genomic DNA library preparation using the Ligation Sequencing Kit V14 (all Oxford Nanopore). We used the 400-bp super accuracy model for basecalling, then performed genome assembly by using Trycycler version 0.5.0 (*26*) as follows. We filtered raw nanopore reads by using Filtlong version 0.2.1 to remove reads of <1,000 bases and the 5% of reads with the lowest quality. We used Trycycler to subsample reads into 12 subsets, then separately reassembled read subsets by using Raven version 1.5.3 (*27*). We used Trycycler to perform clustering, reconciliation, circularization, multiple sequence alignment, and consensus sequence generation from the 12 assemblies. We polished the resulting consensus assembly with nanopore long reads and medaka version 1.4.3 using the r1041_e82_400bps_sup_v4.1.0 model. We aligned Illumina reads to the assembly by using BWA version 0.7.15 (*28*) and corrected assembly errors by using Polypolish version 0.5.0 (*29*) and the POLCA module of MaSuRCA version 4.0.9 (*30*,*31*). We deposited sequences in the NCBI BioProject database (accession no. PRJNA1164417) (Appendix Table 1).

### Single-Nucleotide Variant Identification and Phylogenetic Analysis

We aligned Illumina reads from each isolate to the genome sequence of strain 128-CS by using BWA version 0.7.15 (*28*). We used bcftools version 1.9 and a haploid model to identify single-nucleotide variants (SNVs) and skipped bases with quality <25 or alignment quality <30. We further filtered SNVs by using the bcftools_filter software, as previously described (*32*), to remove variants with SNV quality scores of <200, read consensus <75%, read depth <5, read numbers of <1 in each direction, or locations within repetitive regions as defined by BLAST alignment of the reference genome sequence against itself. We generated a maximum-likelihood phylogenetic tree from the core genome alignment by using IQ-TREE version 2.2.0 and the ModelFinder function to estimate the best fit nucleotide substitution model by means of Bayesian information criterion (*33*,*34*). We used the ultrafast booststrap method (*35*) with 1,000 replicates to assess tree topology. We used phylo-treetime 0.11.4 to estimate time-scaled phylogeny by using the previously inferred maximum-likelihood phylogeny and incorporating specimen sampling dates using the covariation and stochastic-resolve options, an autocorrelated molecular clock with the relax 1.0 0.5 option, and then rerooted the tree by using the least-squares method (*36*). We visualized and annotated the tree in R version 4.2.2 (The R Project for Statistical Computing) by using the ggtree version 3.8.0 and ggtreeExtra version 1.8.1 packages (*37*,*38*).

## Results

**Figure 1:**
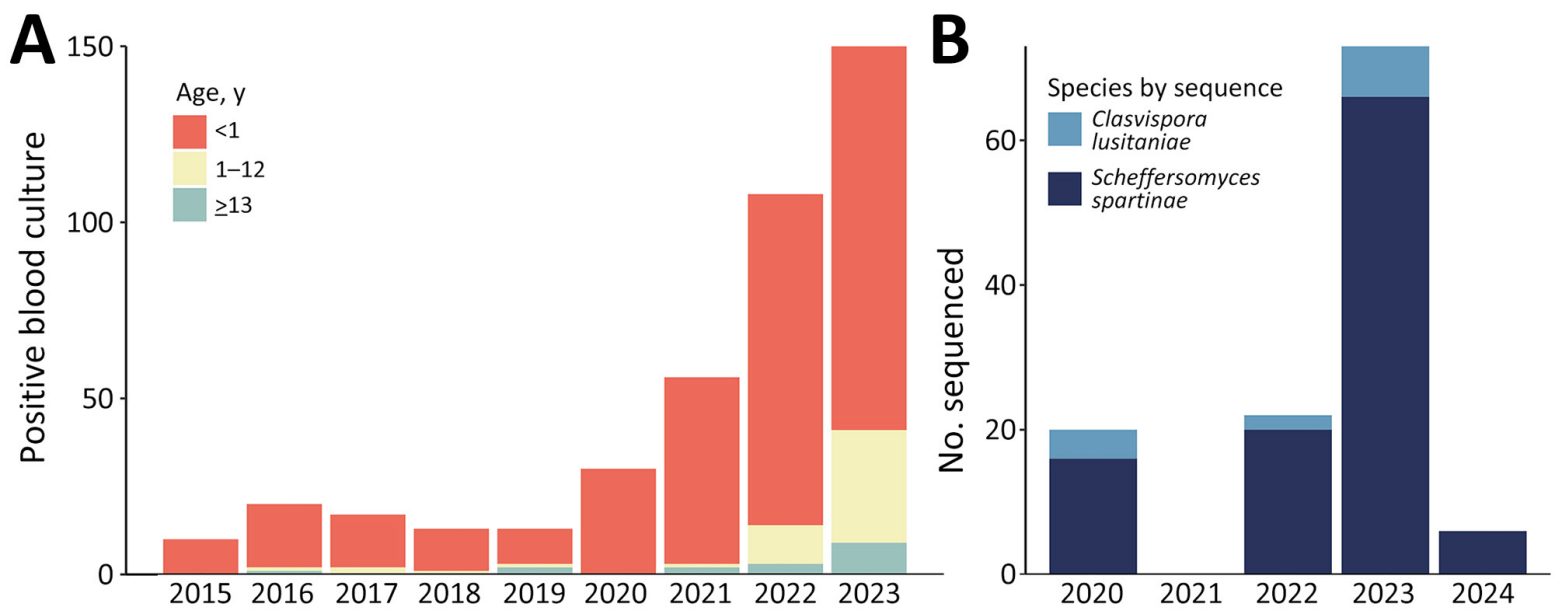
Characteristics of yeast biochemically identified as *Clavispora lusitaniae* during an outbreak of *Scheffersomyces spartinae* fungemia among pediatric patients, Pakistan, 2020–2024. A) Number of blood cultures with yeast identified as *C. lusitaniae* by patient age range and year, 2015–2023. Biochemical species identification performed by VITEK 2 YST ID card system (bioMérieux). B) Species designation by internal transcribed sequence among isolates initially identified as *C. lusitaniae* from blood or other sources that subsequently underwent whole-genome sequencing, 2020–2024.

During 2015–2023, the AKUH clinical microbiology laboratory identified 432 presumed *Clavispora* (formerly *Candida*) *lusitaniae* fungemia cases. Presumed *C. lusitaniae* cases detected from blood cultures increased from 10–20 cases/year during 2015–2019 to 30 cases in 2020 and 56 cases in 2021. The number of cases detected from cultures from blood specimens further increased to 108 in 2022 and to 150 in 2023 (Figure 1, panel A), representing <0.11% of the total annual blood culture specimens processed by the laboratory (Appendix Table 2). Of the 432 *C. lusitaniae* cases, 415 patients (96.1%) were <13 years (4,745 days) of age and 365 (84.5%) were <1 year (365 days) of age: 326 (75.4%) <3 months of age, 274 (63.4%) <1 month of age, and 68 (15.7%) <7 days of age. Reflective of the primary referral base for the AKUH laboratory, most (n = 369; 85.4%) *C. lusitaniae* bloodstream cases were from Karachi, and 241 (55.8%) samples were from 1 tertiary care public sector pediatric hospital.

Using the laboratory practices for isolate banking described herein, 136 presumed *C. lusitaniae* isolates had been banked: 21 of 31 isolates cultured in 2020, 0 of 56 isolates cultured in 2021, 29 of 108 isolates cultured in 2022, 80 of 150 isolates cultured in 2023, and 6 of 15 isolates cultured in January of 2024. To investigate those cases, we performed WGS on banked isolates, 121 of which yielded high-quality assemblies. We excluded 15 isolates because total contig size exceeded 12.3 million bases (n = 8), total contig number exceeded 800 (n = 11), genome coverage was <95% as determined by BUSCO (n = 9), or a combination of those factors. BUSCO analyses showed equivalent rates of assembly completeness, fragmentation, and missingness between the isolates sequenced at either institution. Querying the internal ITS sequences from the whole-genome assemblies against the NCBI ITS database showed that just 13 (11%) of the 121 high-quality genome sequences were true *C. lusitaniae*, whereas ITS sequences from the other 108 (89%) sequences had 99.3%–100% sequence identity with the ITS sequence of *Scheffersomyces spartinae* CBS 6059 (NCBI accession no. NR_111290.1) (Figure 1, panel B; Appendix Table 1).

The median age of *S. spartinae* patients was 19.5 days (range 2 days–12 years); 68.5% of cases were among infants <4 weeks of age (Table 1). For most cases, specimens were obtained in Karachi, reflective of catchment, but >8% of cases were identified in patients from other regions. Most (69.4%) cases were identified at a single hospital (hospital H) in Karachi specializing in neonatal and pediatric care. Blood cultures from that hospital constituted 1.4% of the total blood cultures processed by the AKUH clinical microbiology laboratory since 2020, and 2.9% of blood cultures from that hospital were *C. lusitaniae* positive by biochemical assay (Appendix Table 2). 

**Table 1:** Patient demographic and clinical characteristics from cases of *Scheffersomyces spartinae* fungemia among pediatric patients, Pakistan, 2020–2024

Characteristics	No. (%), n = 108
Age group	
<2 wk	40 (37.0)
2–3 wk	34 (31.5)
1–6 mo	5 (4.6)
7–11 mo	8 (7.4)
1–6 y	14 (13.0)
7–12 y	7 (6.5)
City of residence	
Karachi	99 (91.7)
Lahore	5 (4.6)
Nawabshah	2 (1.9)
Hafizabad	1 (0.9)
Multan	1 (0.9)
Isolation year	
2020	16 (14.8)
2021	0
2022	20 (18.5)
2023	66 (61.1)
2024	6 (5.6)
Hospital or institution	
Hospital B	5 (4.6)
Hospital H	75 (69.4)
Hospital N	5 (4.6)
Hospitals with only 1 case	11 (10.2)
Unknown hospital	12 (11.1)
Clinical details	
Patient family or guardian contacted	92 (85.2)
Hospitalization reported, n = 92	92 (100)
Clinical information available	63 (58.3)
Presumed sepsis, n = 63	43 (68.2)

We cultured *S. spartinae* from specimens received from 14 different institutions but could not determine the origin of 11% of specimens (Table 1). Of patients whose families or guardians could be contacted and consented to provide clinical information (92/108), all were hospitalized at the time of specimen collection. Treating physicians provided admitting diagnoses and brief clinical histories for 63 (58.3%) patients, 43 (68.2%) of whom were admitted for sepsis. MICs for amphotericin, azole, and echinocandin antifungal drugs were low among the 108 *S. spartinae* isolates tested, and MIC_90_ values were <1 µg/mL for all agents in those classes (Table 2).

**Table 2:** MICs of 108 isolates from *Scheffersomyces spartinae* fungemia among pediatric patients, Pakistan, 2020–2024*

Antifungal drug	MIC, µg/mL		
	Detected range	MIC_50_	MIC_90_
Fluconazole	0.06–16	0.5	1
Itraconazole	0.015–2	0.06	0.12
Voriconazole	0.008–1	0.03	0.03
Posaconazole	0.008–1	0.03	0.06
Flucytosine	0.06–64	32	64
Amphotericin	0.03–0.5	0.12	0.25
Caspofungin	0.015–0.5	0.06	0.12
Anidulafungin	0.015–0.5	0.12	0.12
Micafungin	0.015–0.25	0.06	0.12

*MIC_50_, concentration required to inhibit 50% of strains; MIC_90_, concentration required to inhibit 90% of strains.

**Figure 2:**
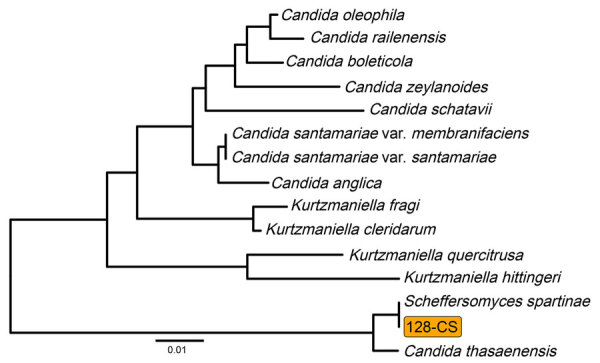
Internal transcribed spacer (ITS) sequence phylogeny of *Scheffersomyces spartinae* isolate 128-CS from fungemia among pediatric patients, Pakistan, 2020–2024. Midpoint-rooted neighbor-joining tree from alignment of ITS sequence of strain 128-CS and the 14 most closely related ITS sequences in the National Center for Biotechnology Information Fungal Internal Transcribed Spacer RNA database (BioProject accession no. PRJNA177353). Scale bar indicates nucleotide substitutions per site.

Isolate 128-CS, which we selected for long-read sequencing, came from a sample collected in Karachi in December 2022. Assembly from long and short reads of 128-CS produced 8 linear chromosomes ranging in length from 1.19 to 2.05 Mbp and 1 circular mitochondrial genome, for a total genome size of 12.27 Mbp (Appendix Table 4). Comparison of the ITS sequence of isolate 128-CS to the NCBI ITS database reconfirmed *S. spartinae* (Figure 2).

**Figure 3:**
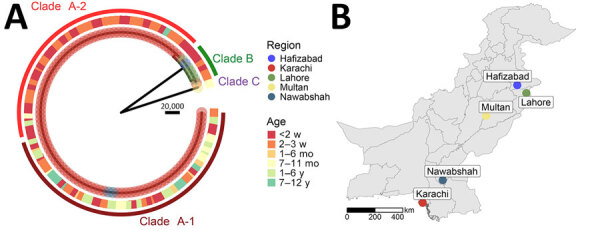
Whole-genome phylogeny and locations of *Scheffersomyces spartinae* fungemia isolates among pediatric patients, Pakistan, 2020–2024. A) Midpoint-rooted maximum-likelihood phylogenetic tree from whole-genome sequence alignment of 108 *S. spartinae* isolates from human blood cultures. Tip circles indicate the patient’s city of residence. Outer squares indicate the age of the patient. Arcs indicate observed major phylogenetic clades or subclades. Scale bar indicates number of single-nucleotide variant differences corresponding to branch lengths. B) Cities of residence for all patients with *S. spartinae*–positive blood cultures detected by whole-genome sequencing.

**Figure 4:**
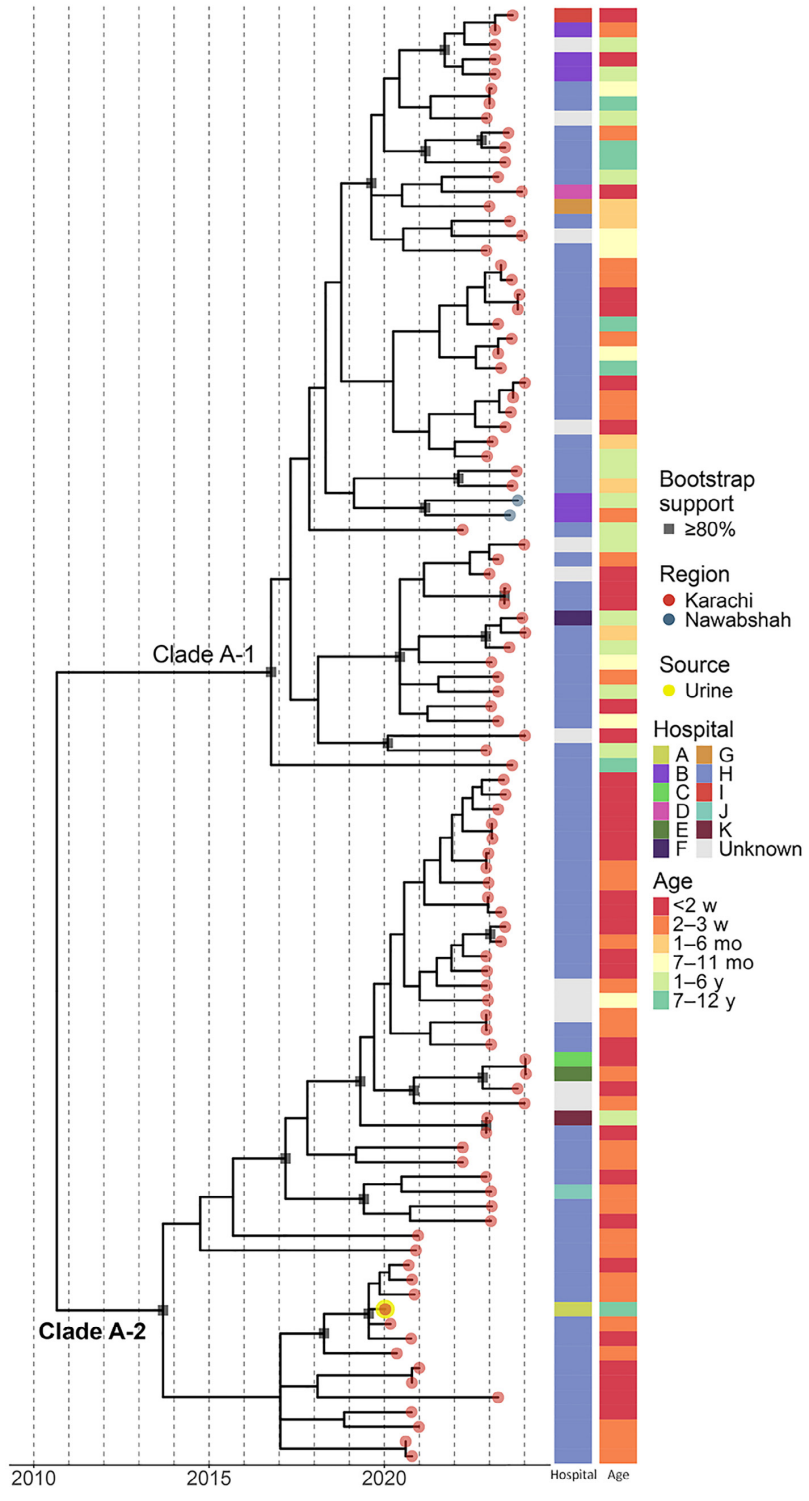
Time-scaled maximum-likelihood phylogenetic tree of *Scheffersomyces spartinae *major clade A in cases of fungemia among pediatric patients, Pakistan, 2020-2024. Nodes marked by gray squares indicate branches with >80% bootstrap support based on 1,000 resamplings. Tree tips correspond to the sampling date. Tip colors indicate patient’s city of residence. All specimens isolated from blood cultures other than the specimen from urine highlighted in yellow. Heatmaps show the anonymized hospital or institution at which the specimen was collected and age group of the patient. Dotted vertical lines represent individual years.

Phylogenetic analysis of all 108 *S. spartinae* sequences aligned to 128-CS revealed that most sequences belonged to a clade consisting of nearly all specimens obtained from Karachi and 2 specimens from Nawabshah (Figure 3). That large clade (clade A) contained 2 subclades, clades A-1 and A-2 (Figure 4). We also identified 2 smaller clades: clade B, consisting of 1 isolate each from Karachi and Hafizabad and all 5 isolates from Lahore, and clade C, consisting of 1 genetically distant sequence isolated from a patient in Multan (Figure 3). Although the Multan isolate differed by >213,000 SNVs from 128-CS, the ITS regions of the 2 isolates were 100% identical. All isolates in clade B were collected from infants <1 month of age; the clade C isolate was collected from a 6-month-old child (Figure 3, panel A).

Because most isolates cultured during 2020–2024 were closely related, we examined the characteristics of those 100 specimens. Within that clade, the median pairwise genetic distance across the 12.2 million base alignment was just 9 (range 0–22) SNVs. The time-scaled maximum-likelihood phylogenetic tree showed that, despite the small genetic distances between sequences, subclades A-1 and A-2 are separated with >80% bootstrap support (Figure 4). Isolates from 2020 were all in clade A-2 and clustered more closely with each other than with most later isolates; we excluded 1 isolate, 2020-498, which did not follow the fitted molecular clock. All 5 isolates from hospital B were in the same subclade; otherwise, we noted no apparent association between sampling location and either isolation date or genetic similarity. 

Isolates from patients <1 month of age predominated in subclade A-2, comprising 93.6% of that clade compared with 38.5% of clade A-1 (p<0.001 by Fisher exact test^­^) (Table 3). No association between patient age and genetic similarity was noted among isolates in either subclade. Only isolates from hospital H were represented in both subclades and were equally distributed (Table 3). Those results suggest that genetic distances are small among *S. spartinae* in that group; that isolates were diverse across time, geography, and patient ages; and that closely related but independent lineages were cocirculating within the same region and institution, potentially indicating separate sources of infection.

**Table 3:** Subclade characteristics from *Scheffersomyces spartinae* fungemia among pediatric patients, Pakistan, 2020–2024*

Characteristics	Clade, no. (%)		p value
	A-1, n = 52	A-2, n = 47	
Age group			
<2 wk	12 (23.1)	23 (48.9)	**0.011**
2–3 wk	10 (19.2)	21 (44.7)	**0.0090**
1–6 mo	5 (9.6)	0	0.058
7–11 mo	6 (11.5)	1 (2.1)	0.11
1–6 y	13 (25)	1 (2.1)	**0.001**
7–12 y	6 (11.5)	1 (2.1)	0.11
Hospital			
A	0	1 (2.1)	0.47
B	5 (9.6)	0	0.058
C	0	1 (2.1)	0.47
D	1 (1.9)	0	1
E	0	1 (2.1)	0.47
F	1 (1.9)	0	1
G	1 (1.9)	0	1
H	36 (69.2)	37 (78.7)	0.36
I	1 (1.9)	0	1
J	0	1 (2.1)	0.47
K	0	1 (2.1)	0.47
Unknown	7 (13.5)	5 (10.6)	0.76

*All p values from Fisher exact test comparing values for each age group or hospital to values from all other age groups or hospitals. Bold font indicates statistical significance.

## Discussion

We describe *S. spartinae* as a cause of fungemia in pediatric patients in Pakistan. *S. spartinae* has been previously described as an environmental organism capable of surviving extreme conditions, but isolation from clinical specimens represents emergence as a human pathogen. The genus *Scheffersomyces* was proposed by Kurtzman and Suzuki on the basis of D1-D2 large subunit rRNA and small subunit rRNA sequencing; species assigned to the *Scheffersomyces* genus were originally included in the genus *Pichia* (*39*). Most *Scheffersomyces* species ferment xylose and are used in industrial applications (*39*–*41*). The assignment of *S. spartinae* to the genus was originally considered uncertain because of weak bootstrap support in the D1–D2 small subunit tree and because *S. spartinae* does not ferment xylose. According to our literature review, *S. spartinae* has not previously been reported as a cause of human infections nor cultured in healthcare facilities. We speculate *S. spartinae* might be emerging in humans because of introduction from environmental sources into healthcare environments. However, given the broad distribution of healthcare facilities from which we cultured *S. spartinae* in Pakistan, we cannot rule out nonhealthcare community sources of infection.

The reasons for the emergence of some fungi as agents of human infection have been multifactorial. Fungi often survive extreme conditions, adapt to selection pressures, and develop enhanced thermotolerance, virulence, and antifungal drug resistance. Emergence of *Candida auris* in human infections has been attributed to global warming (*6*,*7*); some researchers postulate that *C. auris* inhabits an environmental niche and has only recently become a human pathogen (*4*). Low virulence fungi such as *Saccharomyces cerevisiae*, *Saprochaeta clavate*, and *Rhodotorula* spp. can contaminate food, medical products, and healthcare environments and cause infections in susceptible hosts (*10*–*12*). Alterations in the geographic range of *Cryptococcus deuterogattii* from tropical regions to temperate climates have led to human infections in nonendemic areas, attributed to human activity, thermal adaptation, and climate change (*5*). 

Another reason for increased emergence of fungal infections is environmental disruption caused by natural disasters, which can lead to a wider distribution of fungi (*9*). An example is increased human *Coccidioides immitis* infections after earthquake-related landslides and sandstorms in previously low-prevalence areas (*8*). Whether similar or other factors contributed to the emergence of *S. spartinae* as a human pathogen is unclear.

Cases of *C.* *lusitaniae* fungemia identified by the AKUH laboratory began increasing in 2020, and a further substantial rise in cases began in 2022. Most *C. lusitaniae* patients were children <1 year of age who were admitted to hospitals in the city of Karachi. During 2015–2019, rare *Candida* species were reported as causes of invasive infections in neonatal and pediatric populations in Pakistan (*42*). Isolation of *C. lusitaniae* was infrequent, however, so rising case numbers from 2022 onwards were flagged by the laboratory, which more frequently banked cultured isolates. Biochemical identification of *Candida* by the VITEK 2 YST ID card system inaccurately identified the organism, and most of the isolates were ultimately identified as *S. spartinae* by whole-genome and ITS sequencing. The high percentage of isolates subsequently identified as *S. spartinae* suggests that at least some of the isolates identified as *C. lusitaniae* from before 2020 also might have been *S. spartinae*, but we cannot verify that hypothesis because earlier specimens were not banked. 

Species misidentification is concerning and implies that phenotypic identification systems cannot reliably identify emerging pathogens. Misidentification underscores the need for using molecular approaches, such as ITS sequencing, for species identification. Decreasing costs and increasing availability of WGS promise improved species identification as well as strain typing and genomic epidemiology, but the technology remains difficult to access in many low-resource areas.

Although evidence implies that cases of *S. spartinae* infection represent the emergence of a new pathogen, the possibility of a pseudo-outbreak (i.e., clustering of positive cultures because of contamination of clinical specimens incorrectly attributed to infections) must also be considered. Compared with bacterial pseudo-outbreaks, fungal pseudo-outbreaks have rarely been reported. Documented pseudo-outbreaks involving *Candida guilliermondii* were related to inadequate sterile technique during blood culture collection (*43*), contamination of heparin solution used to flush needles before blood draws (*44*), and an anaerobic holding chamber in the clinical microbiology laboratory (*45*). Pseudo-outbreaks reported with other *Candida* species include *C. versatilis* contamination in olive oil used for culture media supplementation (*46*) and *C. parapsilosis* contamination of a salt solution used for grinding tissues (*47*).

Unlike prior pseudo-outbreak reports that involved temporally clustered samples from single institutions, we report isolates collected over the course of 4 years from >14 healthcare institutions across Pakistan. Furthermore, we noted considerable demographic similarity between patients from which the positive specimens were collected: >68% were <1 month of age, and >80% were <1 year of age (Table 1). Although all the isolates were cultured and identified by a single clinical microbiology laboratory at AKUH, all processes and equipment used for processing clinical specimens in the laboratory were independent of the age of the patient from which the specimen was collected. If contamination in the laboratory explained most or all observed cases, we would expect detection in cultures from patients across all age ranges and a larger number of specimens. Even when *C. lusitaniae* cases increased during 2022 and 2023, those cases constituted only 0.57% of 16,505 blood cultures processed from children <1 year of age in 2022 and only 0.60% of 18,051 cultures from children in that age group in 2023 (Appendix Table 2).

The genetic diversity of specimens across and within geographic locations (Figures 3, 4) reduces the likelihood of a single infection or contamination source. Although genetic diversity between isolates within a geographic area of sampling was low, that diversity was not inconsistent with the degree of genetic variation among geographically clustered infections caused by other yeast species, such as *C. auris* (*48*,*49*). Until we learn more about the genomic variability and evolution of *S. spartinae*, the degree of expected genetic difference among epidemiologically linked or unlinked specimens will remain unclear; however, cocirculation of at least 2 lineages in Karachi over the course of >4 years suggests multiple introductions into the region.

One limitation of this study is that, before 2022, AKUH prioritized its finite laboratory resources for investigating and banking invasive antimicrobial-resistant isolates and isolates from known pathogens of epidemiologic concern. As a result, isolates identified as *C. lusitaniae* were not consistently banked, and few isolates from before 2022 were available for species verification and genomic analysis. Another limitation is that detailed patient clinical and demographic information was either unavailable or difficult to obtain because of variability in record-keeping and data availability across the many institutions that provided specimens to AKUH. Many treating physicians could not be contacted because in Pakistan laboratory tests are paid out of pocket by the patients or their guardians, who often provide their own contact details rather than their physicians’ contact information at sample collection. That practice greatly hinders epidemiologic investigations and transmission reconstructions to determine common exposures or shared risk factors among affected persons, to establish patients’ clinical manifestations, and to assess responses to treatments or outcomes. Efforts are ongoing to establish cooperative agreements and study protocols for the most affected institutions in Pakistan for standardized data collection and sharing for *S. spartinae* fungemia cases or other unusual yeasts.

In summary, our findings underscore the value of epidemiologic monitoring for identifying infection clusters and of genomic and molecular surveillance for identifying rare and emerging pathogens. Future studies will be directed at characterizing mediators of pathogenicity and virulence factors that could contribute to *S. spartinae* emergence as a human pathogen, as well as exploring potential environmental reservoirs or other sources of infection. Nonetheless, this study demonstrates that building capacity for specimen identification and banking, WGS, and bioinformatic analysis in low- and middle-income countries like Pakistan is imperative for early detection and study of emerging infectious disease threats. 

Appendix Additional information from *Scheffersomyces spartinae* fungemia among pediatric patients, Pakistan, 2020–2024.
